# Sudden Blast Crisis After Excellent Initial Response in Chronic Myeloid Leukemia

**DOI:** 10.7759/cureus.18368

**Published:** 2021-09-28

**Authors:** Aviraag Vijaya Prakash, Keerthana P Sivakolundu, Natasha M Savage, Vamsi K Kota, Mahran Shoukier

**Affiliations:** 1 Department of General Medicine, Employees State Insurance Corporation Medical College, Bangalore, IND; 2 Department of Hematology and Oncology, Georgia Cancer Center at Augusta University, Augusta, USA; 3 Department of Pathology, Medical College of Georgia at Augusta University, Augusta, USA

**Keywords:** bcr-abl, tyrosine kinase receptor inhibitors, imatinib, blast crisis, rt- qpcr, chronic myeloid leukemia (cml)

## Abstract

Sudden blast crisis is an uncommon phenomenon in chronic myeloid leukemia (CML) patients who are being treated with tyrosine kinase inhibitors (TKIs). Despite well-defined guidelines to treat and monitor the disease, it is difficult to predict the occurrence of a sudden blast crisis. Research directed towards improving guidelines in choosing the appropriate TKIs and better monitoring protocols could help prevent such unfortunate outcomes. We present a case of a 46-year-old man diagnosed with CML who responded well to imatinib as evidenced by a downtrend in quantitative BCR-ABL mutation to less than 1. He quickly transformed into a blast crisis phase after five months of therapy with imatinib regardless of achieving an excellent initial optimal response. In conclusion, it is possible to transform into a blast phase despite achieving an initial optimal response. Therefore, attention should be focused on the selection of proper tyrosine kinase inhibitors and careful monitoring to allow the early detection of sudden blast crisis.

## Introduction

Chronic myeloid leukemia (CML) is a myeloproliferative neoplasm that arises from the abnormal translocation t(9;22)(q34;q11), with shortened chromosome 22 designated as Philadelphia chromosome, 22q. The juxtaposition of the ABL (Abelson murine leukemia), a proto-oncogene on chromosome 9 with the BCR (breakpoint cluster region) gene on chromosome 22, results in an abnormal fusion gene BCR-ABL. This fusion gene codes for BCR-ABL transcripts and fusion proteins that have tyrosine kinase activity [1]. Currently, the mainstay of treatment in CML is the use of tyrosine kinase inhibitors (TKIs), a targeted therapy known to have considerable response rates and benefit in improving the quality of life in CML patients [2].

Imatinib is the first pioneer TKI approved by the Food and Drug Administration (FDA) for the treatment of CML in the chronic phase [3]. Imatinib at a standard 400 mg dose produces early molecular response rates (BCR-ABL1<10% IS (“International Scale”)) between 60-80% at three and six months. Major molecular response rates range between 20-59% and 60-80% at one and five years, respectively [2,4]. The probability of achieving a deep molecular response (DMR) at five years ranges between 35 and 68% [2,4]. The five- and ten-year overall survival rates range between 90-95% and 82-85%, respectively [2]. Among those who did respond, 4-7% either lost response or transformed in the first three years, and these rates drop to 1-2% annually thereafter [5]. The IRIS (International Randomized Study of Interferon versus STI571) study approximates that 17% of the patients on imatinib developed resistance on a five-year follow-up [6]. Owing to the development of resistance or loss of response, newer drugs such as dasatanib [7], nilotinib [5], bosutinib [8], ponatinib [9] have been added to the treatment protocol either to be considered as a first-line therapy or to be used salvage therapy. The rates of change in therapy from imatinib to other TKI, range from 37-50% to 26.5% within five and 10 years, respectively [2].

The European LeukemiaNet guidelines mandate routine monitoring of response to the TKI therapy, with blood counts every two weeks until a complete hematological response is achieved; reverse transcriptase-quantitative polymerase chain reaction (RT-qPCR) on blood cells expressed as BCR-ABL1% according to IS, done every three months; bone marrow cytogenetics by chromosomal banding analysis of marrow cells is useful in monitoring response when used additionally with the above tests but not alone; however, it should be performed when there are atypical translocations or abnormal BCR-ABL1 transcripts which cannot be quantified by qPCR. Atypical transcripts require monitoring with Fluorescence in situ hybridization (FISH) [2].

Although adherence to the above-mentioned protocols likely leads to remission, there is still a probability of failure to respond to treatment. Here, we present a case of a patient diagnosed with CML in the chronic phase who was treated with imatinib. He achieved an initial optimal response evidenced by a BCR-ABL1 qPCR value less than 1 before quickly progressing to an acute leukemoid (lymphoid) blast crisis within five months of treatment.

## Case presentation

A 46-year-old male visited his primary care physician for a history of fever lasting for 14 days in the month of September 2020. It was associated with a 15-pound weight loss, decreased appetite, and weakness. He had a normal leukocyte count two years ago when he underwent a cholecystectomy. Blood work done by his primary care physician revealed an elevated leukocyte count of 30,500 cells/mm^3^ and was hence referred to our hospital for further evaluation.

**Figure 1:**
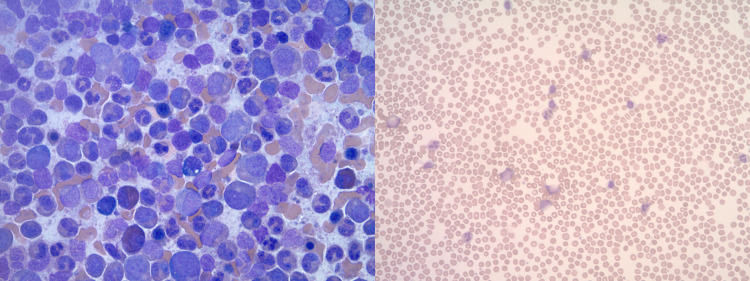
Review of bone marrow aspirate (left) and peripheral blood smear (right) at the time of diagnosis. Aspirate smear shows hypercellular spicules with marked hyperplasia including eosinophilia. Peripheral blood smear shows leukocytosis with neutrophilia granulocyte left shift and basophilia.

Repeat blood work in December 2020 revealed a leukocyte count was 50,700 cells/mm^3^, red blood cell counts 4.6 million cells/mm^3^, platelets count 198,000 cells/mm^3^, absolute neutrophil count, 40,600 cells/mm^3^, basophil count 3500 cells/mm^3^, and a blast count of 500 cells/mm^3^ of blood. The peripheral smear (Figure 1) showed a left shift neutrophilia with myelocytes, metamyelocytes, and rare band forms. Bone marrow aspirate was hypercellular as shown in Figure 1. Cytogenic analysis of cultured bone marrow was performed and analyzed metaphases of all 20 cells revealed 46,XY, presence of Ph chromosome t(9;22)(q34;q11.2) with no additional complex chromosomal abnormalities. FISH revealed a BCR-ABL1 fusion, his bone marrow was hypercellular with myeloid and megakaryocytic hyperplasia, and the percentage of blast cells was 0.3%. A BCR-ABL analysis of his blood sample done by qPCR showed 74489.6 fusion gene (BCR-ABL1) copies and a BCR-ABL1/ABL1 ratio of 55.6640 and was hence diagnosed with chronic myeloid leukemia in the chronic phase.

**Figure 2:**
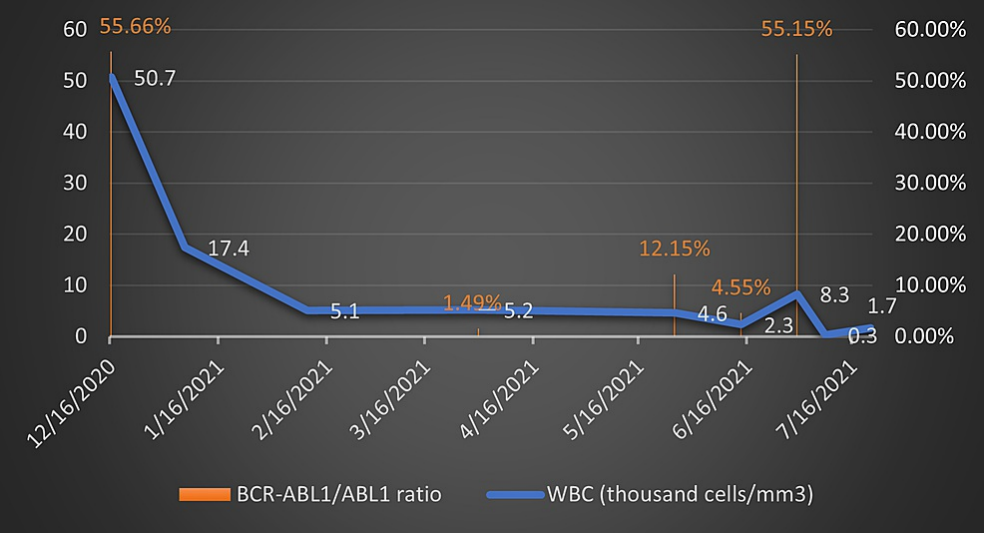
Demonstration of variation of leukocyte counts and BCR-ABL1/ABL1 qPCR analysis with time.

He was treated with allopurinol 300 mg and hydroxyurea to control the white cell count. On January 6, 2021, these medications were discontinued, and the patient was started on imatinib 400 mg once daily. He was followed up weekly and then every month and apart from generalized muscle soreness, which was treated with oxycodone, he tolerated the medication well. His leukocyte counts were monitored during his monthly follow-ups as demonstrated in Figure 2. The patient had an excellent response to imatinib as evidenced by a qPCR done on his blood on March 31, 2021, which showed a reduced fusion gene (BCR-ABL1) copy number of 906, and a BCR-ABL1/ABL1 ratio of 1.4928 (IS ratio: 0.9255).

**Figure 3:**
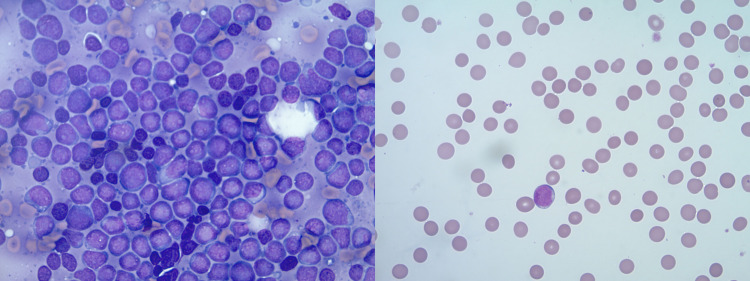
Review of bone marrow aspirate (left) and peripheral blood smear (right) at blast crisis. Aspirate smear shows markedly suppressed trilineage hematopoiesis with predominance of blasts that had morphology similar to peripheral blood. Peripheral smear revealed significant anemia, thrombocytopenia, and neutropenia with many circulating blasts which were medium in size with scant, agranular cytoplasm, and open chromatin pattern with inconspicuous nucleoli.

**Figure 4:**
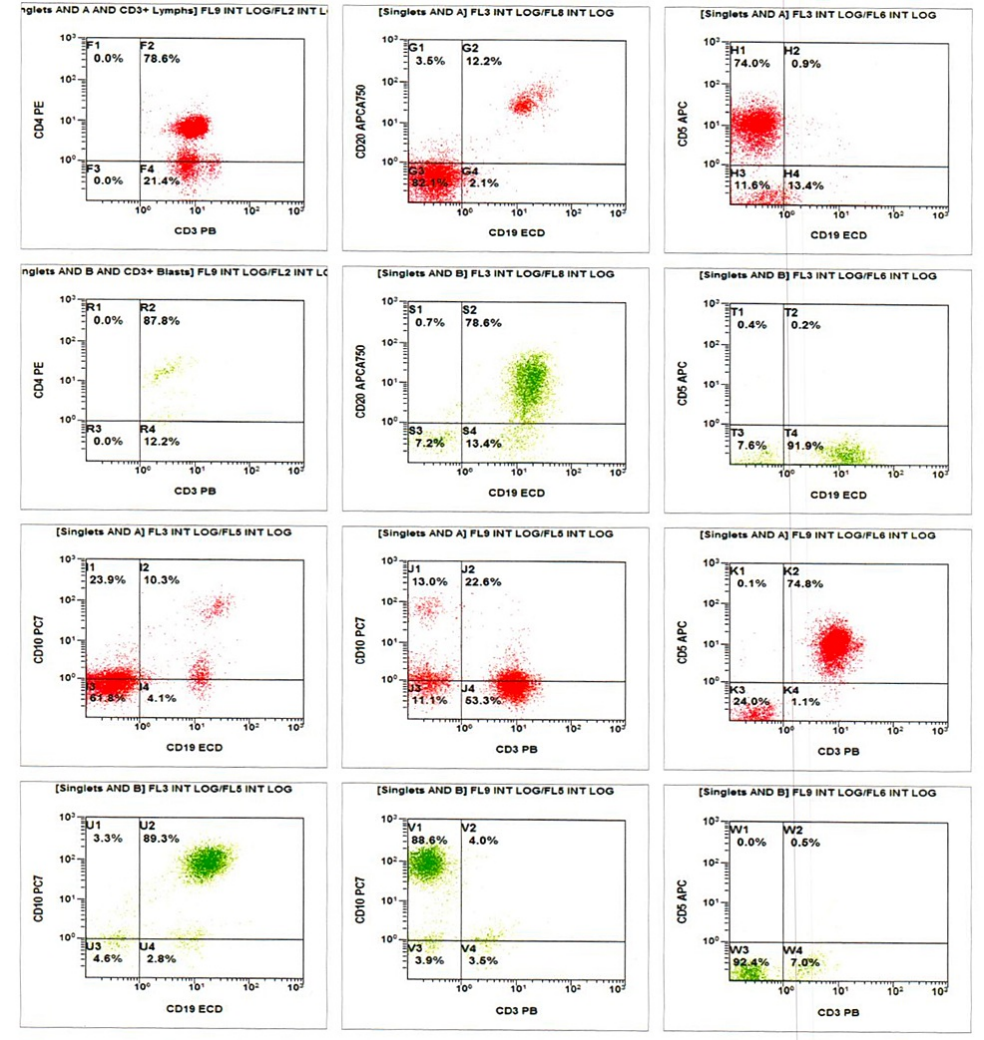
Flow cytometric immunophenotyping revealed a blast population in the CD45 dim gate expressing CD19, CD20 (heterogeneous), and CD10 (bright).

However, after 150 days of treatment, he presented to a local emergency room (ER) with complaints of diarrhea, weakness and he subsequently developed fever, sore throat, and occipital headache. He was found to have leukopenia with an absolute cell count of 1600 cells/mm^3^, and hence was told to hold on his imatinib. RT-qPCR analysis done subsequently showed a fusion gene (BCR-ABL1) copy number of 2646.8 and a BCR/ABL1:ABL1 ratio of 4.5466 (IS ratio: 2.8189). Repeat blood work showed persistent leukopenia and worsening anemia, leading to performance of flow cytometry, which showed an increased B lymphoblast population. Peripheral blood smear showed significant anemia and bone marrow aspirate revealed reduced hematopoiesis with increased blast cells as seen in Figure 3. He was subsequently admitted to the hospital for fever and repeat peripheral blood flow cytometry (Figure 4) showed a lymphoblast population of 35%, which raised concern that he had progressed to blast crisis. The lymphocyte CD45 dim gate revealed a distinct blast population expressing CD20 (dim), CD10 (bright), CD19 (moderate), HLA-DR (bright), and a dim CD38. A repeat qPCR analysis showed a fusion gene (BCR-ABL1) copy number of 114097.5 and a BCR/ABL1:ABL1 ratio of 55.1478.

Due to his persistent headache, and as per CNS prophylaxis management protocol, he was treated with intrathecal cytarabine/methotrexate/hydrocortisone [10]. His TKI was then switched to dasatinib 1450 mg.

## Discussion

With novel procedures and technologies being continuously developed, great advances have been made in the management of CML and many patients are able to discontinue therapy after a period of sustained molecular response [11]. Quantitative PCR analysis has been a successful tool to monitor the targets of therapy [12,13].

Therapy guided by monitoring protocols is based on achieving appropriate response numbers. Prior to TKI therapy, a baseline BCR-ABL1 quantification by RT-qPCR is recommended and following initiation, a three-month follow-up until major molecular response (MMR) is achieved is the protocol. The BCR-ABL1 values attained through RT-qPCR and expressed as “IS” (International scale) for the three, six and 12-month marks are <10%, <1%, and 0.1% respectively, defined by the European LeukemiaNet guidelines [2,14].

It has been shown that high-quality molecular monitoring has helped achieve an appreciable percentage of patients who attain the target milestones and thus maximizing the chance for treatment-free remission [14]. An integrated, closed-loop pharmacy-led chemotherapy program revealed that compliance to therapy is higher in those who are regularly monitored as opposed to those who are not, further highlighting the need for high-quality routine monitoring [15].

Our patient was diagnosed with CML in the chronic phase and underwent the necessary studies required to begin therapy. Cytogenic analysis revealed a translocation t(9;22)(q34;q11.2) with no additional aberrant karyotypes [16]. Following the diagnosis, he was routinely followed up with his leukocyte count on a weekly basis. After he began treatment with imatinib, he was assessed at the three-month mark and was found to have a TKI-sensitive disease and had achieved an optimal response, as evidenced by his BCR-ABL1 (IS) of 0.9255 [17]. Despite achieving this optimal response and being compliant with his treatment strategy, he progressed to blast crisis within five months of treatment with imatinib.

This indicates that, although there is an absence of atypical chromosomal mutations at initial diagnosis, they can be acquired during the pathogenesis or over the course of treatment [18]. This emphasizes the role of routine monitoring as it shifts the standard of management. A baseline and thus subsequent regular quantification of disease burden helps to identify individuals in need of more potent TKIs at the initiation of therapy or in those who have failed treatment with first-line treatment. Failure or resistance to imatinib is not uncommon and has complicated management. This has led to the utilization of newer therapeutic drugs available in the market such as dasatinib [7], nilotinib [5], bosutinib [8], and ponatinib [9]; these target more specific tyrosine kinase domains and are thus more potent and applicable in cases of resistant mutations to imatinib [2]. Ultimately, despite appropriate therapy protocols along with newer and more potent drugs, certain individuals harbor mutations and resistance factors which can lead to adverse outcomes such as sudden blast crisis.

Sudden blast crisis is defined as the development of blast phase CML after a documented complete cytogenic response in the immediately preceding bone marrow analysis and within three months of a normal complete blood count [19]. In patients who develop sudden blast crisis, studies have shown that there is a higher incidence of lymphoid blastic morphology [19]. In the era of TKIs, the development of blast crisis after an optimal response is an uncommon feature but nevertheless a probability. Reports of blast crisis transformation following therapy with imatinib [17,18], dasatinib [20], nilotinib [21,22] show us that there are underlying hidden clinical and biological factors that influence this unfavorable outcome. Ph-positive/Ph-negative mosaicism [17], clonal evolution [18], mutations such as T315l [22] among others are some of the findings observed when the patients transformed into blast phase. It has also been implied that sometimes treatment with TKIs itself provides an opportunity for more indolent and resistant cells to develop. Clinical findings such as red cell distribution width (RDW) have been studied to understand the prognostic value in therapy outcome [23], but despite these rigorous approaches to better understand the disease, we are yet to identify early disease indicators which help us predict treatment outcome and prevent such unfavorable results.

## Conclusions

Anomalies in the natural history of the disease are to be expected and strict emphasis must be placed on following the protocols devised for monitoring and treatment. Nevertheless, the occurrence of such unpredictable events highlights the need to develop improved strategies for identifying hidden disease pathologies. This would help us to not only determine the best initial treatment plan which could possibly lead to an early and more sustained response but would also improve the overall survival rate.

The above literature should call attention to increase research efforts to better understand disease transformation with a focus directed towards the development of molecular markers. This could possibly help us identify resistance factors early in the course of the disease and thereby define prognostic indicators which will aid in therapy and achieve the goal of disease-free survival.
